# Advances in the study of low-density neutrophils in rheumatic diseases

**DOI:** 10.3389/fimmu.2026.1747583

**Published:** 2026-02-27

**Authors:** Jiahua Yin, Qi Wang, Kaiyin Cai, Min Zhang, Xiaoyi Jia

**Affiliations:** 1 School of Pharmacy, Anhui University of Chinese Medicine, Hefei, Anhui, China; 2 Anhui Province Key Laboratory of Bioactive Natural Products, Hefei, Anhui, China; 3 Department of Rheumatology and Immunology, The First Affiliated Hospital of University of Science and Technology of China (USTC), Division of Life Sciences and Medicine, University of Science and Technology of China, Hefei, Anhui, China

**Keywords:** autoimmunity, low-density neutrophils, neutrophil extracellular traps, rheumatic disease, systemic lupus erythematosus

## Abstract

Rheumatic diseases are characterized by immune dysfunction, chronic inflammation and tissue damage, in which neutrophils play a pivotal role. As a heterogeneous subset of neutrophils, low-density neutrophils (LDNs) are present at extremely low levels in healthy individuals, whereas they are abnormally expanded in patients with rheumatic diseases and closely correlated with disease activity. This review summarizes the biological characteristics of LDNs, as well as their roles and potential therapeutic values in various rheumatic diseases. These cells exhibit a dual origin with distinct phenotypic and functional features and exert their pathogenic effects primarily through the release of neutrophil extracellular traps (NETs) and pro-inflammatory cytokines, as well as their involvement in the immune regulatory network. LDNs are implicated in the pathogenesis of multiple rheumatic diseases. Targeting LDNs or their key pathogenic pathways may provide novel insights into the precision therapy of rheumatic diseases. Further investigations into the subpopulations and underlying mechanisms of action of LDNs are therefore warranted to advance the development of targeted therapeutic strategies for these diseases.

## 1 Introduction

Rheumatic diseases are a group of conditions characterized by chronic inflammation, autoimmune responses, and tissue damage, including systemic lupus erythematosus (SLE), rheumatoid arthritis (RA), and antineutrophil cytoplasmic antibody (ANCA)-associated vasculitis (AAV), among others (1). A common feature of these diseases is immune system dysfunction, leading to abnormal activation of immune cells, release of pro-inflammatory factors, and production of autoantibodies, which in turn cause tissue damage and organ dysfunction (2). Neutrophils play a crucial role in innate immune defense and inflammatory responses, additionally regulating adaptive immunity; they have been implicated in the pathogenesis of various rheumatic diseases (3, 4). Neutrophils not only initiate and amplify inflammatory responses but also directly or indirectly cause tissue damage and promote the progression of rheumatic diseases through the release of reactive oxygen species (ROS), neutrophil extracellular traps (NETs), and pro-inflammatory factors (5, 6). In rheumatic diseases, changes in neutrophil numbers, phenotypes, and functions are particularly prominent (7).

**Figure 1:**
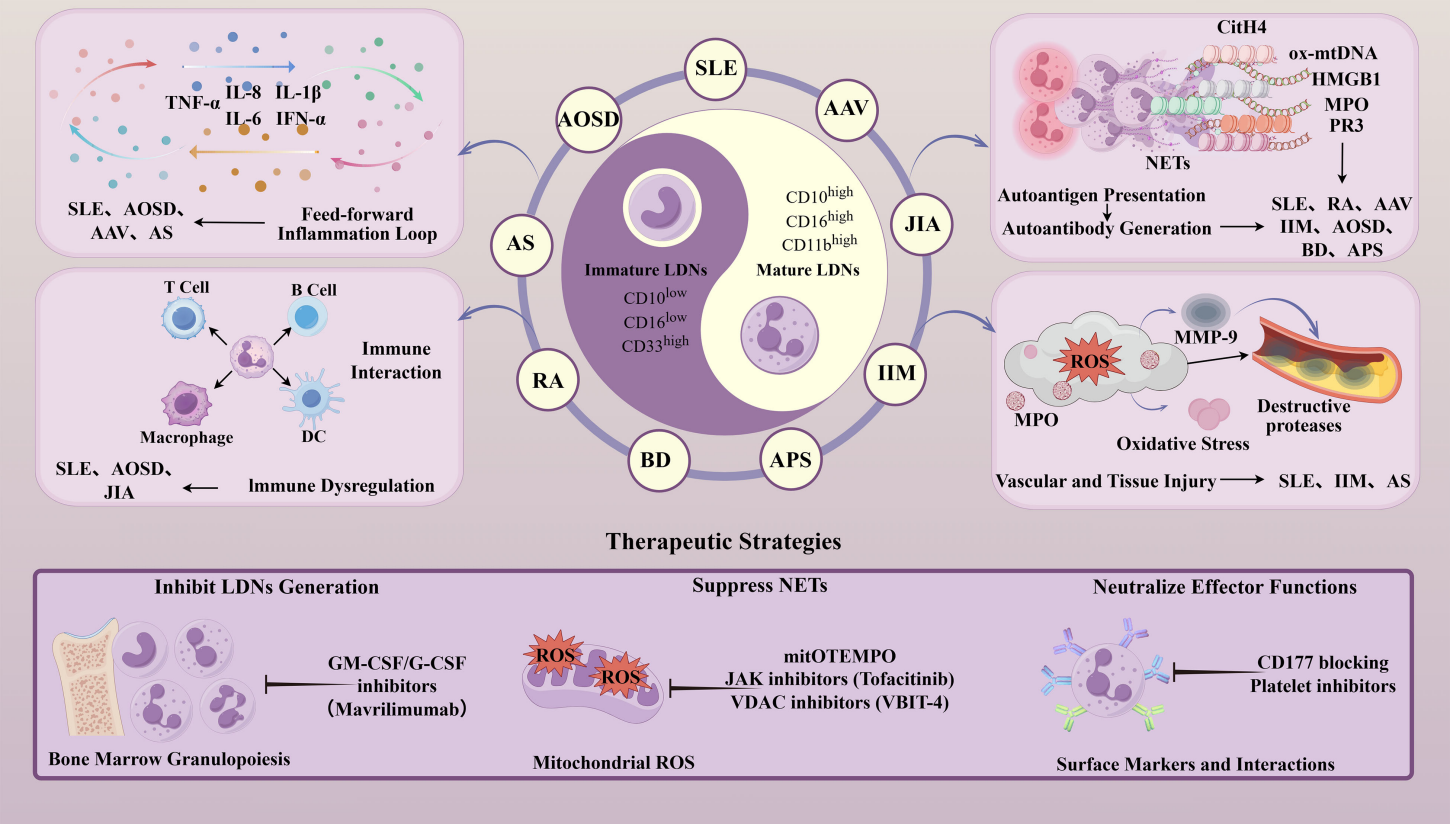
The pathogenic roles of LDNs in rheumatic diseases (SLE, RA, AAV, JIA, IIM, AOSD, BD, AS, and APS) and corresponding targeted therapeutic strategies. By Figdraw. LDNs, Low-density neutrophils; SLE, systemic lupus erythematosus; RA, rheumatoid arthritis; AAV, anti-neutrophil cytoplasmic antibody-associated vasculitis; JIA, juvenile idiopathic arthritis; IIM, idiopathic inflammatory myopathy; AOSD, adult-onset Still’s disease; BD, Behçet’s disease; AS, ankylosing spondylitis; APS, antiphospholipid syndrome; CitH4, citrullinated histone H4; HMGB1, high-mobility group box 1; PR3, Proteinase 3; MPO, myeloperoxidase; ox-mtDNA, oxidized mitochondrial DNA; NETs, neutrophil extracellular traps; ROS, reactive oxygen species.

Low-density neutrophils (LDNs) are a special subgroup of neutrophils. Existing research has shown that they are involved in the pathogenesis of various rheumatic diseases (8–11). LDNs are present in small numbers in healthy individuals but are significantly increased in the peripheral blood of patients with rheumatic diseases and are closely related to the severity of the disease (12, 13). LDNs were first identified in rheumatic diseases, specifically in 1986, when Hacbarth and Kajdacsy-Balla discovered a large number of “low-buoyancy density neutrophils” in the peripheral blood mononuclear cells (PBMCs) of patients with SLE, RA, and acute rheumatic fever (ARF) (14). Based on their lower density compared to normal neutrophils, they termed them “LDNs”. As research has expanded, LDNs have also been detected in the peripheral blood of patients with other rheumatic diseases, such as juvenile idiopathic arthritis (JIA), adult-onset Still’s disease (AOSD), and idiopathic inflammatory myopathy (IIM) (15). In these rheumatic diseases, LDNs exhibit pro-inflammatory characteristics. LDNs can influence the onset and progression of rheumatic diseases by affecting NETs, the production of pro-inflammatory factors, and inflammatory pathways (**Figure 1**). In this review, we describe the biological characteristics of LDNs, as well as their specific roles and potential as a therapeutic target in rheumatic diseases.

## 2 Biological characteristics of LDNs

### 2.1 Isolation and identification of LDNs

It is currently established that LDNs can be isolated from the peripheral blood of humans, rats, mice, pigs, horses and non-human primates (16–20). The isolation protocol for LDNs from human peripheral blood primarily consists of three key steps. The first step in isolating LDNs involves separating PBMCs from peripheral blood using density gradient centrifugation techniques, such as Ficoll-Hypaque or Percoll. Second, LDNs are identified within PBMCs using methods such as Giemsa staining, transmission electron microscopy, and flow cytometry. In flow cytometry, CD14 and CD15 marker expression can distinguish LDNs from monocytes within PBMCs, as LDNs exhibit low CD14 expression and high CD15 expression (21). Third, the LDNs are purified. This can be achieved using flow cytometry or neutrophil immunomagnetic bead sorting to obtain purified LDNs from PBMCs, followed by purity validation to confirm the purity of the purified LDNs (22).

LDNs display low-density characteristics (<1.080 g/mL) due to reduced cytoplasmic granules, decreased nuclear lobulation, or altered membrane components (23). In addition, *in vitro* stimulation with factors such as granulocyte colony-stimulating factor (G-CSF) and certain endotoxins can also induce changes in neutrophil density (24). Density gradient centrifugation is based on the “low density” characteristic of LDNs, whereby LDNs, which are less dense than HDNs, localize to the PBMC layer—the white membranous layer between serum and the liquid surface of Ficoll-Hypaque or Percoll—following density gradient separation, while HDNs precipitate with red blood cells at the bottom of the tube (25). Studies have confirmed that compared to traditional density gradient media involving red blood cell lysis (e.g., Ficoll-Hypaque), the Percoll density gradient method minimizes isolation-induced neutrophil activation and better preserves the cellular response to physiological stimuli, thus being recommended as the method of choice (26). In recent years, researchers have further optimized isolation strategies based on the Percoll method. For example, the use of a negative selection-based immunomagnetic bead isolation kit combined with a discontinuous density gradient medium to isolate LDNs and HDNs from whole blood has enabled high-purity (≥93%) native-state cell isolation (27). In addition, other studies have adopted a CD66b-positive selection-based immunomagnetic bead sorting approach to rapidly isolate LDNs from the PBMC layer after density gradient centrifugation and achieve sorting of LDNs with high-purity (>90%), high-viability (>96%), and intact biological function. Currently, there is a lack of consensus markers for the identification of LDNs (28). While the majority of studies identify LDNs within PBMCs using the CD14^-^CD15^+^ immunophenotypic marker, other studies have also confirmed LDNs in PBMCs using markers including CD66b^+^, CD11b^+^HLA-DR^-^CD66b^+^, CD45^+^CD15^+^ and CD16^+^CD66b^+^, as well as via other methodological approaches (23, 29–32). Such inconsistencies in markers, combined with variations in isolation methodologies, have led to methodological discrepancies in the sorting and functional assessment of LDNs across different studies. At present, the academic community has yet to establish a unified standard regarding the isolation workflow and consensus markers for LDNs. As some researchers have advocated, there is a pressing need to harmonize protocols and research standards for LDNs. Establishing and promoting standardized isolation protocols and research criteria is essential to ensure that results from different studies are directly comparable (33).

### 2.2 Sources and morphological characteristics of LDNs

**Figure 2:**
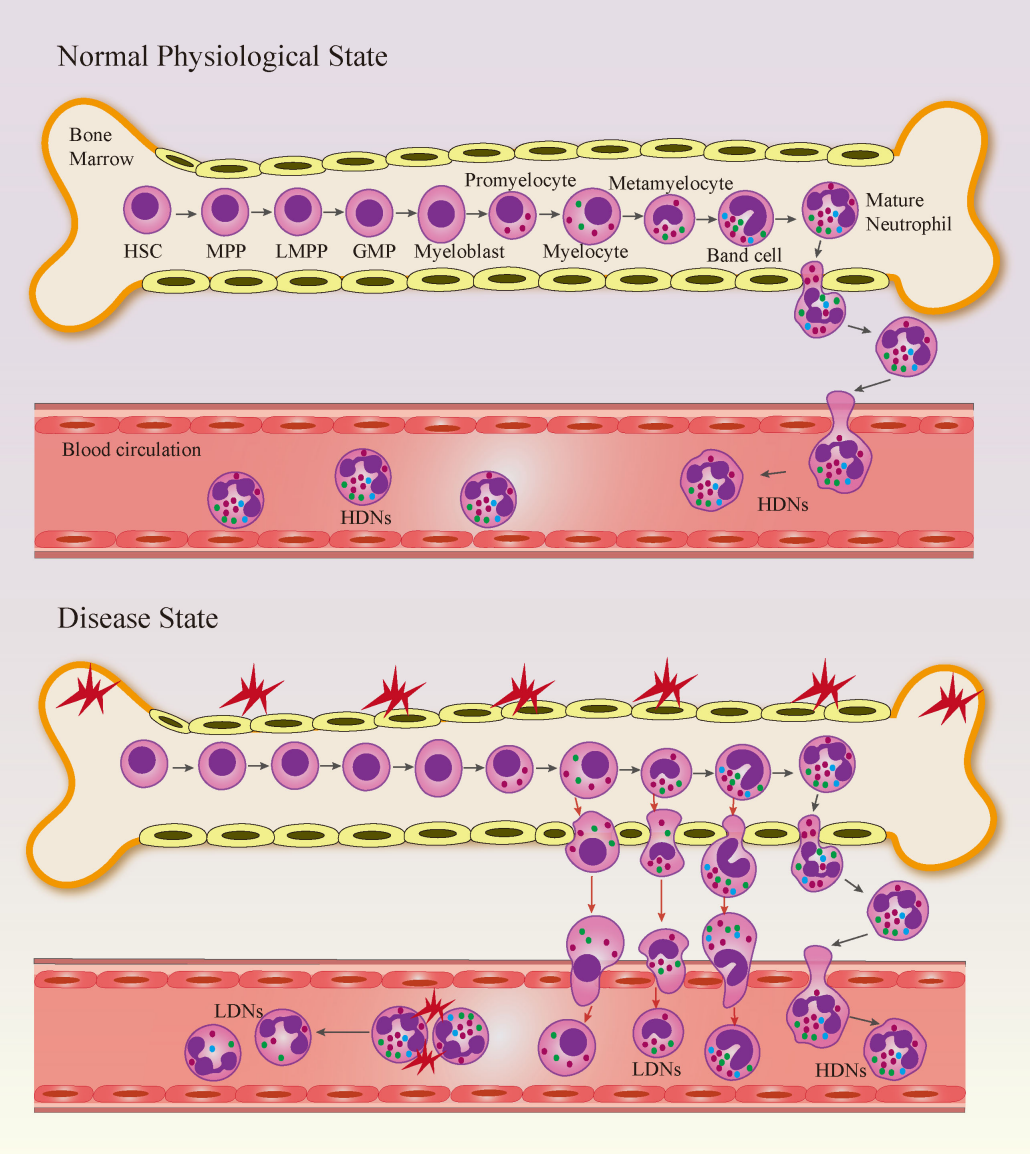
The process of neutrophil maturation and the origin of LDNs. Neutrophils mature in the bone marrow through a stepwise differentiation process: HSCs differentiate sequentially into MPPs, LMPPs, GMPs, and then proceed through various myeloid precursor stages before becoming mature neutrophils. Under pathological conditions, the formation of LDNs occurs via two primary pathways: first, the abnormal release of immature neutrophils from the bone marrow into the peripheral blood, resulting in immature LDNs; and second, the transformation of mature neutrophils in the peripheral blood into mature LDNs through processes such as degranulation following inflammatory stimulation, which reduces their density. LDNs, Low-density neutrophils; HDNs, Normal density neutrophils; HSCs, hematopoietic stem cells; MPPs, multipotent progenitor cells; LMPPs, lymphoid-induced multipotent progenitor cells; GMPs, granulocyte-monocyte progenitor cells.

The dual origin of LDNs is one manifestation of their heterogeneity in rheumatic diseases. Neutrophils mature in the bone marrow, and their production begins with the self-renewal of hematopoietic stem cells (HSCs), which then differentiate into multipotent progenitor cells (MPPs) and lymphoid-induced multipotent progenitor cells (LMPPs). LMPPs further differentiate into granulocyte-monocyte progenitor cells (GMPs), which then undergo myeloblast, promyelocyte, myelocyte, metamyelocyte, band cell, and finally mature stages (34). The production of LDNs can be traced back to two distinct biological processes in the bone marrow and peripheral blood. On one hand, immature neutrophils in the bone marrow are abnormally released into peripheral blood due to granulocyte emergency mobilization in the bone marrow under disease conditions, forming immature LDNs (35). On the other hand, neutrophils in peripheral blood undergo degranulation or other processes under inflammatory stimulation, leading to a decrease in density and transformation into mature LDNs (36, 37) (**Figure 2**). *In vitro* activation and infection assays have provided more direct evidence that mature neutrophils can convert into LDNs under specific conditions (38). Other studies have proposed that LDNs may also be neutrophils in a state of persistent activation or a distinct lineage of neutrophils generated from cells with genomic damage (39, 40). While the origin of LDNs remains a matter of debate, it has been conclusively demonstrated that the release of immature neutrophils from the bone marrow and the activation of mature neutrophils are the predominant sources of LDNs. LDNs derived from these different origins also display characteristic differences in cellular morphology and physical properties. Through Giemsa staining and transmission electron microscopy, it can be observed that the nuclei of immature LDNs are typically rod-shaped or ribbon-shaped, with regular cell shapes, reduced cytoplasmic granules, and abnormal distribution of heterochromatin. In contrast, the nuclei of mature LDNs are lobulated, typically with 2–5 lobes (41, 42). Flow cytometry further revealed that in sepsis, LDNs exhibit higher forward scatter (FSC) than HDNs but similar side scatter (SSC), suggesting their larger cell volume—a phenomenon potentially linked to aquaporin-9-mediated extracellular water uptake by neutrophils, which leads to alterations in cell morphology and density (43). Furthermore, in colorectal cancer, upon subclassification of LDN subsets, it has been found that immature LDNs exhibit significantly lower FSC and SSC values than mature LDNs, whereas mature LDNs and HDNs display comparatively elevated FSC and SSC profiles (44). CD10^+^ LDNs in healthy donors receiving G-CSF therapy exhibit the characteristics of high FSC and low SSC (19). Notably, LDNs from patients with SLE exhibit aberrant accumulation of heterochromatin, a reduction in cytoplasmic specific granules, and the absence of classic apoptotic features (e.g., nuclear fragmentation and cytoplasmic vacuolization) (42). Equine LDNs have a smaller diameter than HDNs, and the proportion of cells with normal nuclear lobulation is lower in the former than in the latter. Collectively, these findings demonstrate that LDNs in distinct contexts show marked disparities in nuclear morphology, cytoplasmic granularity and physical properties, which indicates that divergent pathological microenvironments can drive the differentiation of LDNs into subsets with unique morphological and density characteristics, and this morphological heterogeneity underpins the functional diversity of LDNs.

### 2.3 The function of LDNs

Currently, most studies suggest that LDNs are a heterogeneous group of cells composed of neutrophils at different stages of maturation, including immature and mature neutrophils (45, 46). Across different physiological states, distinct disease conditions, and various stages of the same disease, the ratio of immature to mature neutrophils exhibits significant variations. During pregnancy, mature LDNs exhibit a CD10^+^CD49d^-^Igκ^+^ phenotype and possess typical pro-inflammatory properties, whereas immature LDNs are characterized by a CD10^-^CD49d^+^Igκ^+^ molecular phenotype, display immunosuppressive and proliferative capabilities, and expand during healthy gestation, with the disruption of their balance being closely associated with the onset and progression of gestational diabetes (30). Mature LDNs also serve as the primary immunosuppressive effector cells within the tumor microenvironment, inhibiting T cell proliferation and function through contact-dependent release of arginase-1 (Arg-1), generation of reactive oxygen species (ROS), and LOX-1-mediated signaling pathways (47, 48). In rheumatic diseases, mature LDNs highly express activation markers such as CD10, CD16, and CD11b, indicating their degranulated state and pro-inflammatory nature (49). In contrast, immature LDNs show low expression of CD10 and CD16 but high expression of the immature granulocyte marker CD33 (50). Their functionality is primarily characterized by impairments, such as reduced chemotaxis and defective phagocytic capacity observed in SLE and sepsis, and their accumulation in SLE may reflect increased bone marrow hematopoietic stress and immune dysregulation (51). However, under specific hyperinflammatory conditions like severe COVID-19, certain immature subsets can demonstrate significant procoagulant activity, contribute to immunothrombosis, and display context-dependent pathogenic potential rather than mere functional deficiency (52).

As a key regulatory cell bridging innate and adaptive immunity, LDNs can interact with other immune cells and possess antigen-presenting potential (53). LDNs can form complexes with platelets through P-selectin/PSGL-1 binding and TLR7-mediated pathways, an interaction that not only exacerbates thromboinflammation but also creates a positive feedback loop that promotes the release of NETs (54, 55). The NETs released by LDNs can activate the NLRP3 inflammasome in macrophages and inhibit macrophage polarization toward an anti-inflammatory phenotype by altering ubiquitination modifications, thereby establishing a sustained inflammatory cycle (56, 57). The effects of LDNs on T cells are biphasic. In cancer, sepsis and HIV infection, LDNs suppress T cell function via arginase-1, the PD-L1/PD-1 axis, reactive oxygen species and other mediators (58–62). Recent studies have demonstrated that orbital flight can induce the activation and expansion of LDNs and potentiate their immunosuppressive capacity against T cells (29). In SLE, by contrast, LDNs drive the polarization of CD4^+^ T cells toward the Th1 subset, upregulate the expression of pro-inflammatory cytokines including interferon-γ and tumor necrosis factor-α, and amplify local inflammatory responses (63). LDNs indirectly regulate B cell activation and autoantibody production through the release of NETs and the co-expression of molecules such as ARID3a, whereas B cells can conversely modulate the functions of LDNs via the secretion of regulatory mediators, which together drive immune dysregulation and pathological damage (64–67). In addition, in patients with incomplete SLE, MPO-DNA complexes, a core marker associated with LDN-derived NETs, are positively correlated with the proportions of age-associated B cells and memory B cells, and negatively correlated with the proportion of naive B cells (68). In terms of antigen presentation, within an inflammatory microenvironment containing GM−CSF and IFN−γ, LDNs can upregulate the expression of MHC class II molecules and co−stimulatory molecules CD80 and CD86, and also possess the antigen processing and presenting capacity, enabling effective antigen presentation to CD4^+^ T cells and driving their differentiation toward Th1/Th17 phenotypes, with some LDN subsets capable of cross−presenting exogenous antigens (69–73).

### 2.4 LDNs in tissues and tissue fluids

Currently, research on LDNs is no longer limited to peripheral blood. Increasingly, researchers are focusing on their presence, phenotypic characteristics, and functional roles in specific tissue microenvironments and tissue fluids. For example, studies have detected LDNs in the postoperative peritoneal lavage fluid of gastric cancer patients and demonstrated that their released NETs can mediate tumor cell growth and adhesion (74). Similarly, infiltrates of mature LDNs have been identified in colorectal cancer tissues, metastatic liver tissues and peritumoral liver tissues in colorectal cancer (44). In the lung parenchyma of COVID-19 patients, substantial infiltration of LDNs (CD11b^+^CD66b^+^CD16^int^) has been observed (75). Furthermore, LDNs have been detected in the synovial fluid (SF) of patients with acute gout (76). This also provides direction for future research, namely, combining the peripheral blood, the tissue, and the tissue fluid samples from patients to explore the specific role of LDNs in the disease. Next, we will introduce in detail the specific mechanism of action of LDNs in the disease.

## 3 The role of LDNs in rheumatic diseases and their mechanisms

Although LDNs originate from neutrophils with key defensive functions, their numbers are significantly increased in various rheumatic diseases, and they exhibit strong pro-inflammatory properties, potentially mediating excessive inflammatory responses and tissue damage. The following will focus on typical rheumatic diseases such as SLE, RA, and AAV to explain the role and mechanism of LDNs in rheumatic diseases.

### 3.1 LDNs in SLE

SLE is a chronic autoimmune disease of unknown etiology, whose pathogenesis involves abnormalities in innate and adaptive immunity triggered by a combination of genetic and environmental factors (77, 78). The levels of LDNs in the peripheral blood of SLE patients are significantly elevated and are significantly correlated with the disease activity index (SLEDAI) score, serum complement levels, and anti-dsDNA antibodies (79). Functional defects in the U12-type minor spliceosome in LDNs lead to aberrant splicing of immune regulatory genes such as CYBA and reduced NADPH oxidase activity, and these defects are closely associated with disease activity and autoantibody profiles (80). LDNs play a key role in the pathogenesis of SLE, with their mechanisms of action involving the differentiation of heterogeneous subpopulations, the formation of NETs, the activation of type I interferon signaling, and a complex network of immune-mediated damage to multiple organs.

LDNs exhibit a unique pro-inflammatory phenotype in the peripheral blood of SLE patients. Based on differences in CD10 expression, LDNs can be divided into two functionally distinct subpopulations. CD10^+^ LDNs exhibit a mature multinucleated morphology, highly express genes of the type I interferon pathway (ISG15, MX1) and pro-inflammatory factors (IL-6, IL-8, TNF-α), and possess enhanced NET formation capacity, directly damaging endothelial cells by releasing MMP-9 and oxidized mitochondrial DNA (ox-mtDNA) (54). In contrast, CD10^−^ LDNs exhibit immature nuclear morphology, high expression of cell cycle-related genes (CDK2/4/6), and promote CD4^+^ T cells to produce IFN-γ and TNF-α, but have impaired immune function, suggesting that they may originate from the abnormal release of immature granulocytes from the bone marrow (8). In patients with active SLE, the proportions of CD177^+^ neutrophils and CD177^+^ LDNs are markedly elevated and positively correlated with disease activity, and the specific targeting of the CD177^+^ neutrophil subset may provide a novel therapeutic avenue for SLE treatment (81).

In this disease, ox-mtDNA and citrullinated histone H4 (CitH4), released within NETs from mature LDNs, not only act as self-antigens to activate the TLR9 pathway in B cells—thereby driving anti-dsDNA antibody production—but also stimulate the NLRP3 inflammasome in SLE macrophages, triggering pyroptosis and enhancing IL-1β and IL-18 secretion; these cytokines in turn promote further NET formation, while LDN-derived NETs (LDN-NETs) themselves potently induce IFN-α release (56). IFN-α further enhances LDNs’ ability to form NETs in a feedback loop and inhibits endothelial repair functions, creating a sustained inflammatory feedback loop (51). In other words, LDNs amplify autoimmune responses through a NETs-IFN-α vicious cycle (82).

In terms of organ damage, LDNs participate in the pathological process of SLE through multiple mechanisms. In the cardiovascular system, MMP-9 released by LDNs can disrupt vascular endothelial cell junctions, while NETs formed by LDNs can oxidize high-density lipoprotein, causing it to lose its anti-atherosclerotic function (83). Additionally, LDNs-platelet complexes promote thrombosis in lupus nephritis, while extracellular CitH4 may further induce pyroptosis in vascular smooth muscle cells by inducing pyroptosis and chronic inflammation, thereby accelerating the instability of atherosclerotic plaques (84). In terms of kidney injury, NETs deposited in glomeruli activate the complement system through LL-37-DNA complexes, recruiting inflammatory cells to infiltrate and form a vicious cycle, while also promoting renal interstitial fibrosis by activating Snail protein to drive endothelial-mesenchymal transition (82). Furthermore, reduced DNase I activity in the serum of SLE patients further impairs NETs clearance, exacerbating kidney injury (85). In neuropsychiatric SLE, LDNs may promote neuroinflammation through multiple mechanisms, including the production of pro-inflammatory cytokines and the release of factors that disrupt the blood-brain barrier, such as MMP-9 and neutrophil gelatinase-associated lipocalin (86). These findings systematically elucidate the key mechanisms by which LDNs contribute to multi-organ damage in SLE.

**Figure 3:**
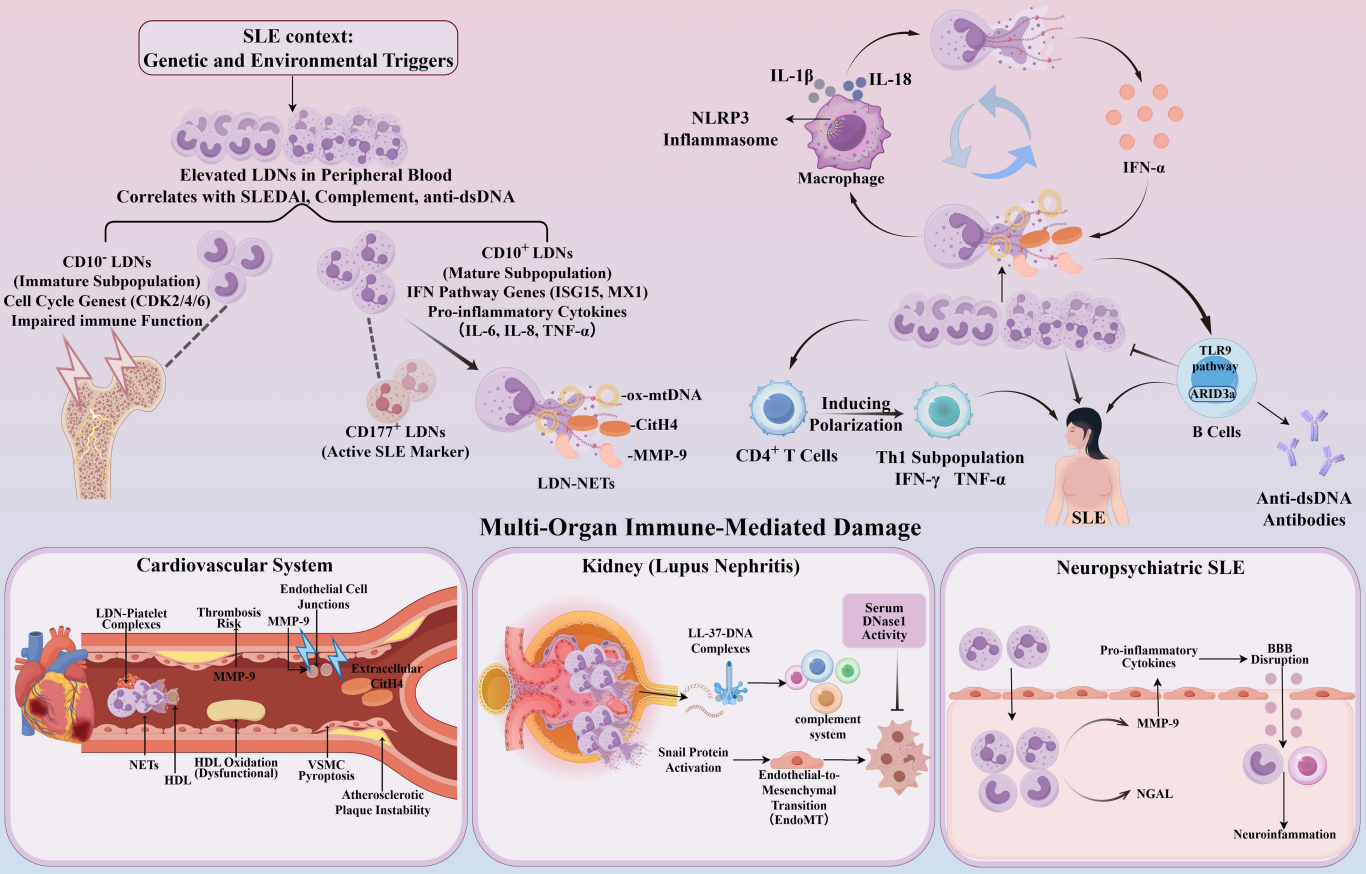
The pathogenic roles of LDNs in the pathogenesis and progression of SLE and the mechanisms of multi-organ damage. By Figdraw. LDNs, Low-density neutrophils; SLE, systemic lupus erythematosus; NETs, neutrophil extracellular traps; IL-1β, Interleukin-1β; IL-18, Interleukin-18; IFN-α, Interferon Alpha. IFN-γ, Interferon gamma; TNF-α, Tumor Necrosis Factor alpha; MMP-9, Matrix Metalloproteinase-9; HDL, High-Density Lipoprotein; VSMC, Vascular Smooth Muscle Cell; CitH4, Citrullinated Histone H4; NGAL, Neutrophil Gelatinase-Associated Lipocalin.

LDNs play a central role in the pathogenesis of SLE through mechanisms such as heterogeneous subpopulation differentiation, the NETs-IFN-α vicious cycle, and multi-organ immune injury networks (**Figure 3**). LDNs in SLE patients and the free DNA they release are not only important indicators of disease activity but also powerful biomarkers for predicting the risk of cardiovascular and skeletal complications (87). Targeting LDN-platelet interactions and inhibiting NET formation may provide new intervention targets for SLE treatment.

### 3.2 LDNs in RA

RA is a chronic systemic autoimmune disease characterized by persistent synovitis, systemic inflammation, and continuous production of autoantibodies (88). LDNs exhibit unique phenotypic characteristics and functional abnormalities in the pathogenesis of RA, with their numbers positively correlated with the disease activity score (DAS28). However, they exhibit significant differences in functional characteristics compared to HDNs and LDNs in SLE patients. LDNs in RA patients exhibit contradictory phenotypic characteristics. Although their CD10 expression levels are similar to those in HDNs, they exhibit low CD16 expression, active cell cycle gene expression and a lack of response to TNF-α (89). This mismatch between phenotype and function reflects disrupted granulocyte differentiation in the pathological state of RA, suggesting that LDNs in RA may be a mixed population consisting of mature neutrophils and immature neutrophils.

Functionally, LDNs in RA patients exhibit significant defects, including diminished chemotaxis, phagocytosis, and ROS production, while maintaining the ability to form NETs both spontaneously and upon Phorbol-12-myristate-13-acetate (PMA) stimulation at levels comparable to neutrophils (89). NETs have been shown to promote autoantibody production and joint damage in RA by exposing citrullinated antigens and activating fibroblast-like synovial cells (90, 91). Transcriptomics and qPCR analyses confirmed that the mRNA levels of myeloperoxidase (MPO) and NE in LDNs were significantly higher than those in HDNs, suggesting that RA-LDNs may participate in the pathogenesis of RA through the NETs pathway (89). The unique phenotypic characteristics and functional defects of LDNs in RA patients reflect the regulation of neutrophil differentiation and function by the RA-specific pathological microenvironment.

### 3.3 LDNs in AAV

AAV is a group of systemic autoimmune diseases characterized by ANCA targeting neutrophil granule proteins MPO and Proteinase 3 (PR3), leading to small vessel inflammation and organ damage (92, 93). LDNs, a key pathological cell subset in AAV, consist of immature CD10^-^LDNs and mature CD10^+^LDNs (46). In patients with active AAV, the proportion of LDNs in peripheral blood is significantly elevated and exhibits notable heterogeneity: approximately 75% are terminally differentiated mature neutrophils, while 25% exhibit features of immature neutrophils, such as incomplete nuclear lobulation; this immature subset is characterized by low expression of CD16, CD10, and CXCR2, and high expression of CXCR4 (94). Additionally, active-phase patients exhibit high expression of granulocyte-related genes MPO and PR3, which are positively correlated with disease activity and significantly upregulated in treatment-resistant patients (95).

Similar to LDNs in SLE, the immature CD10^−^ LDNs subpopulation in AAV patients exhibits kidney-shaped nuclei and high expression of DEFA1, suggesting that this subpopulation may originate from prematurely released granulocytes from the bone marrow (72). LDNs in AAV patients exhibit unique pro-inflammatory characteristics and functional defects, potentially contributing to disease pathology through the release of inflammatory factors and metabolic alterations. Although IL-8 secretion by LDNs is lower than that of HDNs in the resting state, ANCA stimulation significantly increases IL-8 release from LDNs; this enhanced IL-8 further recruits neutrophils to inflammatory sites, forming a positive feedback loop that exacerbates vascular inflammation (96). LDNs exhibit significantly reduced phagocytic capacity for fluorescently labeled Escherichia coli particles compared to HDNs and neutrophils from healthy volunteers, potentially due to metabolic dysfunction and low expression of CD16 (96). The core pathological role of LDNs is manifested in their spontaneous formation of NETs, which release self-antigens such as PR3 and MPO; this process not only directly damages the vascular endothelium but also promotes ANCA production (97). Functionally, immature CD10^-^ LDNs highly express self-antigens but do not respond to antigen stimulation, while mature CD10^+^ LDNs participate in pathogenesis through NETs (46). The expression of self-antigens in HDNs is directly correlated with the intensity of ANCA-mediated oxidative bursts, making them the primary effector cells in vascular damage (98). At the genetic level, LDNs upregulate pro-inflammatory genes such as IL-8, TNF, and IL-1β, while downregulating metabolic pathway genes such as PDK1 and GLUT1 (96). Currently, there are limited biomarkers capable of predicting treatment response in AAV patients, while elevated levels of LDNs in AAV are associated with severe symptoms and reduced therapeutic response, suggesting that LDNs and LDNs-related genes may serve as potential biomarkers for disease activity and treatment outcome.

### 3.4 LDNs in JIA

JIA is a common connective tissue disease in childhood, characterized primarily by chronic arthritis, which may be accompanied by systemic multi-organ involvement (99). In the pathogenesis of JIA, LDNs exhibit significant increases in number and functional abnormalities, participating in the development and progression of the disease (100). The proportion of LDNs in PBMCs from JIA patients is significantly higher than in healthy controls, and it correlates positively with serum calcitonin gene-related protein (S100A8/A9) levels, suggesting that LDNs may serve as potential markers of inflammatory activity. These LDNs exhibit immature neutrophil characteristics (low expression of CD10) and an activated state (low expression of CD62L), with significantly reduced levels of surface markers CD66b and CD11b. This unique phenotypic characteristic may be associated with the premature release of granulocytes from the bone marrow driven by inflammatory factors such as G-CSF and TNF-α (11). Additionally, LDNs in JIA patients exhibit significant functional abnormalities. The levels of proteases they release (e.g., MMP-8 and LF) are elevated, directly contributing to joint inflammation and tissue damage (101).

Moreover, LDNs in JIA patients exhibit impaired immune regulatory function, with reduced ability to inhibit T cell proliferation and decreased PD-L1 expression, potentially leading to an imbalance in immune tolerance. Although there are no significant differences in LDNs proportions among different JIA subtypes (e.g., oligoarticular, polyarticular, and enthesitis-related arthritis), CD62L expression is negatively correlated with the number of inflamed joints and disease activity scores (JADAS), suggesting that LDNs may migrate from inflamed joints into the circulatory system via “reverse migration” and participate in local inflammatory cascades in joints (11). Compared with other rheumatic diseases such as SLE, LDNs exhibit unique biological behavior in JIA, providing a theoretical basis for the development of targeted therapeutic strategies.

### 3.5 LDNs in IIM

IIM is a heterogeneous group of autoimmune diseases characterized by chronic muscle inflammation and muscle weakness, often accompanied by myositis-specific antibodies and extra-muscular organ damage (e.g., interstitial lung disease, ILD) (102). In the peripheral blood of IIM patients (especially adult dermatomyositis), the number of LDNs is significantly elevated, with levels comparable to those in SLE, and positively correlated with disease activity (103). LDNs in IIM possess the ability to spontaneously form NETs, a characteristic enabling their direct involvement in pathological damage; these LDN-derived NETs, containing CitH4, can directly induce muscle tubule damage *in vitro* and drive autoimmune reactions through the exposure of self-antigens such as ox-mtDNA (104). The levels of LDNs are positively correlated with the severity of skin disease (e.g., skin ulcers, calcifications) and negatively correlated with muscle strength (104). In patients with dermatomyositis complicated by ILD, LDNs levels are abnormally elevated, suggesting that LDNs may serve as a potential biomarker for ILD (105). RNA sequencing of muscle biopsies revealed significantly elevated expression of neutrophil-specific genes in muscle biopsies from IIM (especially dermatomyositis) patients, and this feature was positively correlated with the type I IFN pathway, suggesting that LDNs may activate the type I IFN pathway through NETs, exacerbating muscle tissue inflammation (104). Therefore, LDNs release pathogenic NETs (containing CitH4 and ox-mtDNA) through spontaneous NETs, directly damaging muscle tissue, amplifying type I IFN inflammatory signals, and being closely associated with extra-muscular complications (e.g., ILD), confirming their core pathogenic role in IIM.

### 3.6 LDNs in AOSD

AOSD is a systemic inflammatory disease characterized by dysregulation at the intersection of innate and adaptive immunity, typically presenting with high fever, arthritis, salmon-colored rash, leukocytosis, and multi-organ involvement (106, 107). During the course of AOSD, the proportion of LDNs in the circulation is significantly increased, and these cells exhibit a highly pro-inflammatory state (108). Studies have shown that LDNs levels in active AOSD patients are significantly higher than those in remission patients and healthy controls, and are positively correlated with disease activity (e.g., CRP, ESR, clinical scores), and decrease after disease remission (109). LDNs exert their core pathogenic role through the spontaneous formation of NETs, which are rich in high-mobility group box 1 (HMGB1) and CitH4. On the one hand, they directly activate vascular endothelial cells and macrophages, triggering systemic inflammation (e.g., bursts of IL-1β and TNF-α). On the other hand, HMGB1 in NETs acts as a damage-associated molecular pattern, promoting autoimmune responses through the TLR4/RAGE receptor signaling pathway and disrupting immune tolerance (110). Notably, further studies reveal that LDNs are an important cellular source of IL-6 in AOSD patients, with their IL-6 production capacity significantly higher than that in healthy individuals, and circulating LDN levels highly correlated with serum IL-6 levels (10). Clinical studies have confirmed that the levels of NETs formed by LDNs are positively correlated with AOSD disease activity (e.g., systemic scores and CRP) and the severity of characteristic skin rashes. Combined with the finding that LDNs are the primary cell source of IL-6, this suggests that LDNs and the LDN-NETs axis it mediates are key mechanisms driving pathological damage in AOSD.

### 3.7 LDNs in other rheumatic diseases

In Behçet’s disease (BD), the number of LDNs in peripheral blood is significantly increased, and these cells exhibit unique functional characteristics. Although their phagocytic capacity and ROS production are reduced, their ability to form NETs is markedly enhanced (111). LDNs participate in anti-infective immune defense by releasing NETs and can also exacerbate vascular inflammatory responses by activating vascular endothelial cells and promoting platelet aggregation, which is closely associated with the characteristic vascular inflammatory lesions (e.g., retinal vasculitis) and thrombosis in BD (112, 113). From a cellular composition perspective, the LDNs population in BD patients exhibits high heterogeneity, including both immature granulocytes and potentially granulocyte-like myeloid-derived suppressor cells (G-MDSCs) with immune regulatory functions. Nevertheless, overall, they primarily exhibit a pro-inflammatory phenotype (111).

Additionally, in ankylosing spondylitis (AS), immature granulocytes (IG) constitute an important component of LDNs, with their peripheral blood proportions significantly elevated and closely correlated with disease activity (114). Clinical observations have shown that IG levels in AS patients are positively correlated with inflammatory markers (CRP, ESR) and disease activity scores (BASDAI), and they decrease significantly after effective biologic therapy, suggesting that IG may serve as a biomarker for disease activity and treatment response. In terms of pathogenic mechanisms, IG in AS patients can participate in the formation of a chronic inflammatory microenvironment by enhancing ROS production and the release of pro-inflammatory factors, thereby promoting the pathological processes of synovial inflammation and bone joint damage. Since IG can be quantitatively analyzed through routine blood tests, they have good clinical accessibility, making them a potential practical indicator for monitoring AS disease progression (114).

In antiphospholipid syndrome (APS), LDNs are significantly increased in patients’ peripheral blood and exhibit unique pro-inflammatory characteristics. Compared with HDNs, LDNs have a higher basal activation state (increased expression of CD11b and CD66b) and stronger NET formation capacity, but lower responsiveness to external stimuli (115). In APS, the increase in LDNs is associated with anti-β2 glycoprotein I antibodies, which can directly induce NET release, unlike the association between LDNs and interferon characteristics in SLE (79). LDNs participate in the thrombotic mechanism of APS by releasing NETs, which not only provide a scaffold for thrombi but also expose self-antigens to maintain autoimmune responses. Studies indicate that APS patients have a higher proportion of activated neutrophils (CD62L^low^/CD16^high^) in their LDNs, and their enhanced NET formation capacity may be a key factor contributing to thrombotic tendencies (115). These findings suggest that LDNs may not only serve as biomarkers for inflammatory states in rheumatic diseases but also potentially as therapeutic targets.

## 4 Therapeutic targeting of LDNs

Although biologics targeting B cells, T cells, or cytokines have significantly improved the efficacy of treatments for rheumatic diseases such as SLE and RA, a significant proportion of patients still face challenges such as inadequate treatment response, persistent disease activity, or recurrence (116, 117). Recent studies have revealed that LDNs are a group of immune cells with unique pathogenic characteristics that play a key role in driving inflammation, tissue damage, and NETs-related autoimmunity in rheumatic diseases. However, existing therapies struggle to effectively target these critical pathogenic populations. Therefore, developing treatment strategies that specifically target LDNs has become a key to breaking through current treatment bottlenecks and achieving precise intervention.

In rheumatic diseases, interventions can be focused on two main aspects: inhibiting the production of LDNs and interfering with their key functions. G-CSF is a key factor in regulating the production of neutrophils in the bone marrow (118). G-CSF knockout mice exhibit a significant inhibition of neutrophil production in the bone marrow, thereby blocking the onset of collagen-induced arthritis, while the anti-GM-CSF receptor monoclonal antibody Mavrilimumab has demonstrated efficacy in RA. Its mechanism of action may involve targeting the G-CSF/GM-CSF signaling pathway to reduce LDNs levels by decreasing the release of immature neutrophils from the bone marrow, thereby exerting an anti-RA effect (119). Furthermore, in a pristane-induced lupus model, LDNs release NETs during the early stages of disease onset. NETs provide autoantigens, activate the IFN pathway, and directly trigger autoimmune reactions and organ damage. This finding provides a critical theoretical basis for the notion that early diagnosis and targeted treatment of SLE can be guided by LDN-NETs (120). Although the mitochondrial ROS scavenger mitoTEMPO lacks specific targeting ability for LDNs, it effectively reduces the formation of LDN-derived NETs by broadly inhibiting mitochondrial ROS-dependent NETs, thereby improving kidney damage in lupus-prone mice (121). In another study, the voltage-dependent anion channel (VDAC) oligomerization inhibitor VBIT-4 significantly suppressed LDN-induced NET formation in lupus-prone mice by blocking mitochondrial membrane pore formation, thereby reducing the release of mtRNA and mtDNA and alleviating disease progression (122). In SLE patients, the formation of NETs induced by IFN-α and SLE serum was significantly reduced after pretreatment with JAK inhibitors (123). This suggests that JAK inhibitors may alleviate the pathological process of SLE by inhibiting NET formation and type I interferon production. The JAK inhibitor tofacitinib significantly reduces LDNs levels in SLE patients, with more pronounced effects in patients carrying the STAT4 risk allele (124, 125). Furthermore, it is important to note that LDNs exhibit prominent sex-specific metabolic and phenotypic differences, and the glycolytic propensity of LDNs in females may further exacerbate the chronic inflammatory process by enhancing the release of NETs and pro-inflammatory cytokines, a feature that also highlights the potential value of LDNs as a therapeutic target for rheumatic diseases with a higher prevalence in females (126). Therefore, the development of therapeutic strategies that specifically target LDNs represents a key approach to breaking through current therapeutic bottlenecks and achieving precise intervention.

## 5 Conclusion

LDNs, as an important neutrophil subpopulation in rheumatic diseases, offer valuable insights into disease pathogenesis when studied in depth. From their initial discovery in the peripheral blood of patients with SLE, RA, and ARF, to their current identification in autoimmune diseases, infectious diseases, and tumors, in which abnormal increases in their numbers and dysfunction are closely associated with disease severity, LDNs have emerged as key cells linking innate and adaptive immunity. Their cellular heterogeneity and distinct functional patterns not only present research challenges but also provide potential opportunities for precision therapeutic interventions. Future studies employing single-cell multi-omics and functional assays will be essential to dissect LDN subsets, elucidate their plasticity, and identify disease-specific pathways. Targeting LDNs represents a promising frontier for improving outcomes in patients with rheumatic diseases.

## Glossary

AAV: anti-neutrophil cytoplasmic antibody-associated vasculitis
ANCA: antineutrophil cytoplasmic antibody
AOSD: adult-onset Still’s disease
aPL: antiphospholipid antibodies
APS: antiphospholipid syndrome
ARF: acute rheumatic fever
AS: ankylosing spondylitis
BD: Behçet’s disease
CitH4: citrullinated histone H4
DAS28: disease activity score in 28 joints
FSC: forward scatter
G-MDSCs: granulocyte-like myeloid-derived suppressor cells
GMPs: granulocyte-monocyte progenitor cells
HDNs: High-density neutrophils
HSCs: hematopoietic stem cells
IFN-α: Interferon Alpha
IFN-γ: Interferon gamma
IG: immature granulocytes
IIM: idiopathic inflammatory myopathy
IL-1β: Interleukin-1β
JIA: juvenile idiopathic arthritis
LDNs: Low-density neutrophils
LMPPs: lymphoid-induced multipotent progenitor cells
MPO: Myeloperoxidase
MPPs: multipotent progenitor cells
NETs: neutrophil extracellular traps
NLRP3: he Pyrin Domain Containing Protein 3
ox-mtDNA: oxidized mitochondrial DNA
PBMCs: peripheral blood mononuclear cells
JADAS: juvenile arthritis disease activity score
PMA: Phorbol-12-myristate-13-acetate
PR3: Proteinase 3
RA: rheumatoid arthritis
RBC: Red Blood Cell
ROS: reactive oxygen species
SF: synovial fluid
SLE: systemic lupus erythematosus
SLEDAI: Systemic Lupus Erythematosus Disease Activity Index
SSC: side scatter
TLR4: Toll Like Receptor 4
VDAC: voltage-dependent anion channel
